# The combination of PKM2 overexpression and M2 macrophages infiltration confers a poor prognosis for PDAC patients

**DOI:** 10.7150/jca.38981

**Published:** 2020-02-03

**Authors:** Hai Hu, Wenzhi Tu, Yungu Chen, Ming Zhu, Huan Jin, Ting Huang, Zhengzhi Zou, Qing Xia

**Affiliations:** 1 Department of Oncology, State Key Laboratory for Oncogenes and Related Genes, Renji Hospital, School of Medicine, Shanghai Jiaotong University, Shanghai Cancer Institute, Shanghai, 200127, China; 2 The Comprehensive Cancer Center, Shanghai General Hospital, Shanghai Jiao Tong University School of Medicine, Shanghai 201620, China; 3 Department of Obstetrics and Gynecology, Shanghai General Hospital, Shanghai JiaoTong University School of Medicine, Shanghai 201620, China; 4 MOE Key Laboratory of Laser Life Science & Guangdong Provincial Key Laboratory of Laser Life Science, College of Biophotonics, South China Normal University, Guangzhou, Guangdong 510631, China.

**Keywords:** PDAC, macrophage, PKM2, prognosis

## Abstract

Macrophages play a critical role in the initiation and progression in various human solid tumors; however, their role and transformation in pancreatic ductal adenocarcinoma (PDAC) were still illusive. Here, immunohistochemistry was used to determine CD206 (specific marker of M2 macrophage) and PKM2 expression in PDAC tissues. Statistical analysis, such as Pearson χ^2^ test, Spearman's rank test, Kaplan-Meier and COX regression assay were used to evaluate their roles on PDAC prognosis. Data showed that both CD206 and PKM2 were elevated and responsible for a poor prognosis for PDAC. In addition, we showed that the two factors were positively correlated; co-overexpression of the two factors conferred the worst prognosis and functioned as an independent prognostic factor for the disease. Our data showed that M2 macrophage infiltration was correlated with PKM2 expression in PDAC cells. The two markers exerted synergistic effect on PDAC progression. Our results suggested dual-target inhibition M2 macrophage polarization and PKM2 expression of cancer cells might be novel approaches to treat PDAC.

## Introduction

Pancreatic ductal adenocarcinoma (PDAC) is a lethal disease with the worst prognosis among major human malignancies with the 5-year survival rate of less than 5%, and the median survival time of 6 months 1. Tumor distant metastasis and chemoresistance are two major causes for chemotherapy failure of PDAC 2. In the recent years, tremendous progress had been obtained toward the diagnosis and treatment of PDAC in preclinical research; however, little treatment from preclinical research had been translated to serve the patients in the clinics. Herein, it is of great necessity to explore the neoplastic machinery so as to reveal novel therapeutic targets.

Rapid progress has been made in researches toward the genomics and proteomics of PDAC 3, 4; however, it brings few benefits in the clinical settings 4. The microenvironment within PDAC is significantly different from that in normal pancreatic tissues. Tumor microenvironment has been reported to contribute the development of various human cancers. Tumor microenvironment functions as the “soil” for the neoplastic cells, and it is very complex and composed of multiple types of cells, chemokines and cytokines 5. Macrophage which is derived from circulating monocytes and responsible for homeostasis, is one of the most abundant leukocytes within the microenvironment 6. Macrophages can be categorized into two distinct subtypes: M1 and M2.M1 macrophages (so called classically activated macrophages) are triggered by Th1-related cytokines and bacterial products, and inhibit tumor development. M2 macrophages (so called alternatively activated macrophages) are activated by Th2-related factors, and facilitate tumor progression 7. The two macrophages mutual convert with the change of microenvironment. The existing evidences document that macrophages in human cancers are mostly M2 subtype and contribute tumor chemoresistance, angiogenesis and distant metastasis. Previous study has showed that the degree of infiltrating macrophages responsible for aggressive behaviors in PDAC 8. However, little was known about the prognostic value and mechanisms of M2 macrophage polarization in PDAC.

O'Neill et al. showed that pyruvate kinase M2 (PKM2), an indispensable member of pyruvate kinase family, can attenuate LPS-induced proinflammatory macrophage, while promoting M2 macrophage polarization 9. It suggested a positive correlation between aerobic glycolysis and M2 macrophage. Additionally, previous study also showed that macrophage usually undergone genetic reprogramming toward M2 upon aerobic glycolysis so as to adapt the fast change of microenvironment 10. Apparently, these data suggested that PKM2 was effective in M2 macrophage polarization. In the present study, the role of M2 macrophage (characterized by CD206 positive) and the potential polarization mechanism was examined in PDAC. The data showed that high M2 macrophage infiltration was an independent prognostic factor for PDAC. Meanwhile, the data also showed that PKM2 expression was upregulated, and PKM2 can function as an independent prognostic factor in PDAC. Finally, we showed that CD206 expression was positively correlated with PKM2 expression. Moreover, simultaneous high expression of PKM2 and CD206 predicted the worst prognosis in all PDAC patients.

## Materials and Methods

### Cell culture

The PDAC cell lines Capan-2 and Aspc-1 were obtained from the American Type Culture Collection (ATCC). Cells were grown in DMEM medium (Gibco, Life Technologies, Carlsbad, CA) supplemented with 10% (v/v) fetal bovine serum (FBS) (Gibco) at 37°C in 5% CO2 incubator. Cells were grown in monolayer and passaged routinely 2-3 times a week. All cell lines were validated by STR fingerprinting and were routinely screened for mycoplasma. Peripheral blood mononuclear cell (PBMC) from the buffy coats of healthy donors (the Renji Hospital) was obtained by density gradient centrifugation with lymphocyte isolation solution (Qiagen). Monocytes were isolated from PBMC cells by positive magnetic separation using CD14 immunomagnetic beads (Miltenyi Biotec, Bergisch, Germany). CD14+ cells (10^6^/mL) were cultured in 1640 media with 10% FBS in 48-well flat-bottom culture plates. The adherent monocytes were incubated for 7 days in RPMI-1640 medium supplemented with 50 ng/mL of M-CSF (Peprotech Inc., Rocky Hill, NJ, USA) to become macrophages. The fresh growth media with same concentration of M-CSF was replaced every 2 days for a total of 7 days.

### Plasmid transfection and drugs treatment

PKM2 shRNA and overexpression plasmids were purchased from Addgene. These plasmids (2μL) were transfected with 2μLs of Lipofecta-mine® 3000 (Thermo). TEPP46 were purchased from SIGMA. Prior to drugs treatment, cells were incubated for at least 12 h and thereafter replaced with media containing drugs; DMSO-treated cells were used as a mock control. Cells were treated with 5μM of TEPP46 for 48h.

### Patients

All 77 patients with PDAC were included in our study. The paraffin-embedded surgical tissues, together with the adjacent tissues were collected from the department of pathology of Shanghai General Hospital from 2012 to 2014. The last follow-up visit was on February28^th^, 2017. The patients' clinical pathological parameters included age, gender, TNM stage, primary tumor location, nerve invasion, vascular invasion, and nuclear grade (Table-1). Each patient provided written informed consent. The Ethics Committees of Shanghai General Hospital approved the study. The criteria for the inclusion were listed: 1. Radiologically, patients' primary tumors originate from the pancreas, and they had undergone the surgery; 2. Pathologically, the specimen after the surgery had been diagnosed as pancreatic cancer; 3. Patients must provide signed informed consent for research.

### Tissue microarray construction

The tissue microarray was made as described previously 11.

### Immunohistochemistry (IHC)

The standard protocol for IHC had been described previously 12. Briefly, the microarrays were dewaxed and dehydrated in xylene and alcohol solutions in sequence. Endogenous peroxidase activity was blocked using 0.3% hydrogen peroxide for 10 mins, before antigen retrieval was undertaken by putting the slides in 0.01 M citrate buffer (pH 6.0) at 98 °C for 5 mins using a microwave oven. The slides were cooled to room temperature and blocked by incubating with normal goat serum at room temperature for 1h, followed by incubation at 4 °C overnight with primary antibodies (CST, Beverly, MA, USA). Finally, the sections were incubated with HRP-labeled secondary antibody and visualized using diaminobenzidine.

### Evaluation of PKM2 staining

Evaluation of PKM2 staining was performed by two independent pathologists blind to the study in five areas at 400× magnification. The staining was scored according to the staining intensity and percentage. Staining intensity was assigned as 0 (no), 1 (weak), 2 (moderate), and 3 (strong). The staining percentage was classified into four categories: 1 (≤25%), 2 (25%-50%), 3 (50%-75%), and 4 (75%-100%). The final score was calculated as staining intensity × percentage. For statistical analyses, a score <6 was treated as negative, and >6 was positive.

### The evaluation of macrophages infiltration

TAMs were defined as cells with membranous staining in the stroma. The section was scanned at low magnification (×100) to identify areas with the greatest number of macrophages. Macrophage density was estimated (per mm2) at a higher magnification (×400). The density was classified into: 0, (<20 macrophages); 1, (between 20 and 40 macrophages); 2, (between 40 and 60 macrophages); and 3, (>60 macrophages). For statistical analysis, they were divided into low (0-1) and high (2-3) infiltration.

### Co-culture system with macrophages and PDAC cells

PBMC-derived macrophages were co-cultured for an additional 72 h with PDAC cells to generate TAMs. Macrophages were seeded in upper inserts of 6-well transwell plate [0.4μm pore size polycarbonate transwell filters (Corning BV Life Sciences, Schiphol-Rijk, The Netherlands)], and cancer cells were seeded in lower inserts. The two cells were co-cultured without direct contact. After 48 h of co-culture, the macrophages in the upper inserts were discarded, and breast cancer cells were used for next treatment.

### Western blot analysis

Cell extracts were prepared using lysis buffer (50 mM Tris-HCl, 150 mM NaCl, 1 mM EDTA, 0.1% SDS, 0.5% deoxycholic acid, 0.02% sodium azide, 1% NP-40, 2.0 mg/mL aprotinin, 1 mM phenylmethylsulfonylfluoride). The cell lysates were centrifuged at 12,000 rpm for 30 min at 4 °C, and then were collected. The protein concentration was determined by Bradford dye method. Equal amounts (30 μg) of protein were subjected to electrophoresis and run in 10% sodium dodecyl sulfate-polyacrylamide (SDS-PAGE). Then proteins were transferred to PVDF membranes (Millipore) for antibody blotting. The membranes were blocked with 5% non-fat milk for 1 h at room temperature, and then incubated with PKM2 and Actin antibodies purchased from Cell Signaling Technologies (Massachusetts, USA). Subsequently, the membranes were incubated with a HRP-conjugated secondary antibody (Protein Tech Group, Chicago, IL) at room temperature for 1 h. The signals were stimulated with Enhanced Chemiluminescence Substrate (GE Healthcare; Munich, Germany), according to the manufacturer's instructions 13.

### Flow cytometry

Staining for CD206 and CD163 were performed with CD206-APC, CD206-PE, CD86-PE and CD163-FITC antiboides (eBioscience) by Flow cytometry (FACScalibur) using the CellQuestPro software. The analysis was performed on nonadherent macrophages harvested by washing the peritoneal cavity with 5ml of sterile NaCl 0.9%. Collected cells were centrifuged at 1,500 RPM for 10 min and the cell pellet was suspended in PBS medium supplemented with 1% fetal calf serum (FCS). Surface expressed CD86, CD206 and CD163 was detected, respectively and was compared with an irrelevant appropriate isotype control.

### Statistical analysis

Statistical analysis was performed using SPSS software (version 21.0; SPSS Inc., Chicago, IL, USA). The relationships between the clinical pathlogical factors and the expression of PKM2 andCD206 were investigated using Pearson χ^2^ test. The Spearman's rank test was used to evaluate their correlation. Kaplan-Meier and COX regression assay were used to evaluate the prognostic value of the clinical pathlogical parameters. *P*<0.05 was considered statistically significant.

## Results

### Clinical pathlogical parameters of the patients

All of the PDAC patients included 22 males and 25 females with their ages ranged from 14 to 75 (mean age, 33.6). For clinic stage, 73 patients were diagnosed as stage I and/or II, while 4 were diagnosed as metastatic disease. The primary tumor sites of the cancers were head and neck (n=40), followed by the body and tail of pancreas (n=37). The summary of other parameters of is shown in Table-1.

### M2 macrophages infiltrated extensively in PDAC

**Figure 1:**
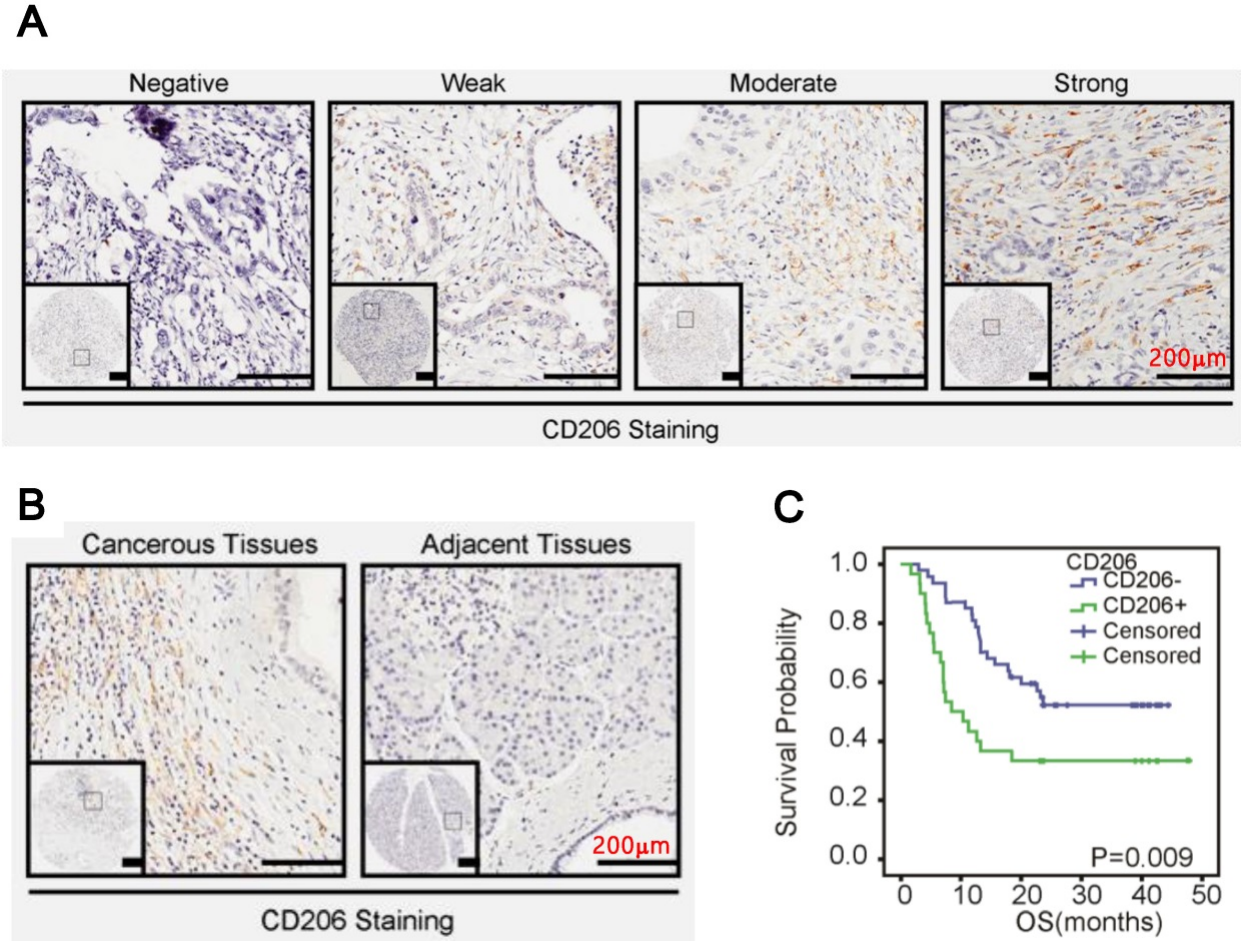
CD206 was upregulated in PDAC. A. Representative images of CD206 expression in PDAC. B. CD206 expression in the cancerous tissues and paired normal tissues. C. Kaplan-Meier survival curves of patients with PDAC. OS based on levles of CD206 expression in PDAC.

We investigated the biological significance of M2 macrophages (characterized by CD206 positive) in PDAC. As shown in Figure 1A, M2 macrophage infiltration in PDAC ranged from negative to strong, with strong staining in predominance. Additionally, macrophages in the cancerous stroma were significantly higher than in the noncancerous counterparts (Figure 1B, p=0.03, Table 2). Statistically, macrophages infiltration was positively correlated with clinic stage (p=0.0473, Table 3) of PDAC. Furthermore, Kaplan-Meier and the COX regression analysis indicated that high M2 macrophage infiltration conferred a poor prognosis (Figure 1C, p=0.009) and functioned as an independent prognostic factor for the patients (95% CI: 1.154-2.976, p=0.0106, Table 5).

### PKM2 was overexpression in PDAC

**Figure 2:**
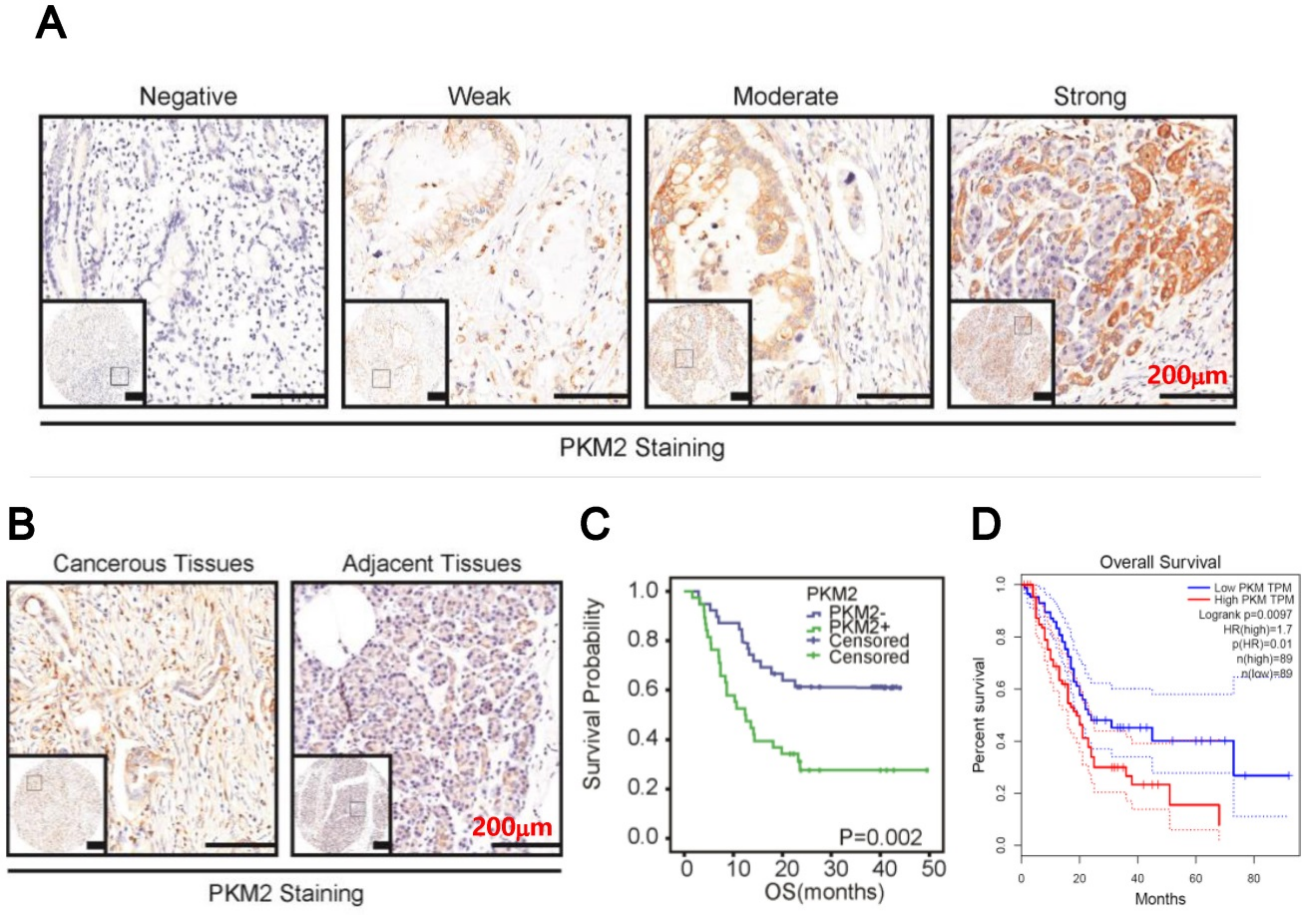
PKM2 was overexpression in PDAC. A. Representative images of PKM2 expression in PDAC. B. PKM2 expression in the cancerous tissues and paired normal tissues. C. Kaplan-Meier survival curves of patients with PDAC. Overall survival (OS) based on PKM2 expression in PDAC. D. Overall survival based on PKM2 expression in PDAC from TCGA database by GEPIA online analysis.

We then examined PKM2 expression in PDAC. As shown in Figure 2A, PKM2 staining ranged from negative to strong, with strong staining in predominance. Statistically, PKM2 staining in the cancerous tissues was significantly higher than in the paired normal tissues (Figure 2B, p=0.0322, Table 2). Then, data also indicated that PKM2 expression was positively correlated with TNM stage (p=0.0002, Table 3) and vascular invasion (p=0.012, Table 3), but not other parameters. Finally, the survival analysis in combined with COX regression assay indicated that PKM2 overexpression conferred a poor prognosis (Figure 2C) and might be an independent prognostic factor for the disease (95% CI: 1.309-3.426, p=0.0022, Table 5). In addition, by analysing TCGA database using GEPIA online software, we found PKM2 overexpression conferred a poor prognosis in PDAC (p<0.01, Figure 2D).

### The combination of PKM2 and CD206 expression is an independent prognostic factor for PDAC

**Figure 3:**
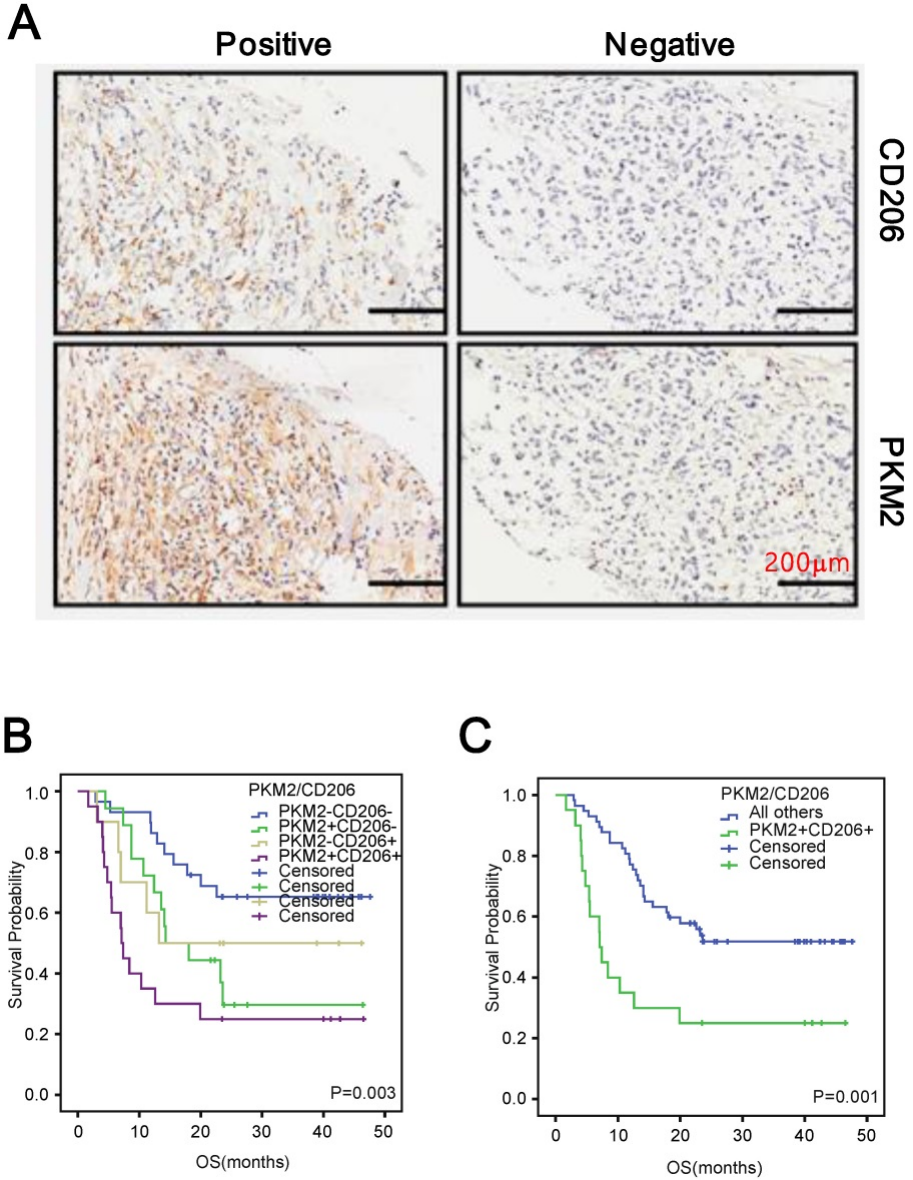
Positive correlation between CD206 and PKM2 in PDAC. A. Representative images depicted the positive correlation between CD206 and PKM2 in the series sections of PDAC. B and C. Kaplan-Meier survival curves of patients with PDAC. OS based on CD206 and PKM2 expression in PDAC.

Since both PKM2 overexpression and high M2 macrophages infiltration were pro-tumoral in PDAC, we examined whether they had synergic effect on PDAC survival. To this end, we initially examined whether PKM2 expression and M2 infiltration were correlated in PDAC. We observed that PDAC tissues with PKM2 positive staining accompanied with high M2 macrophages infiltration (Figure 3A). More importantly, a positive correlation could also been observed between the two factors (r=0.175, p=0.03, Table 4). The Kaplan-Meier analysis in combined with the COX regression assay indicated that simultaneously high PKM2 expression of the cancer cells and M2 macrophages infiltration conferred the worst prognosis (Figure 3B and C), which also functioned as an independent prognostic factor for the patients (95% CI: 1.956-5.735, p=0.001, Table 5).

### Overexpression of PKM2 in PDAC promotes macrophage toward M2 polarization

**Figure 4:**
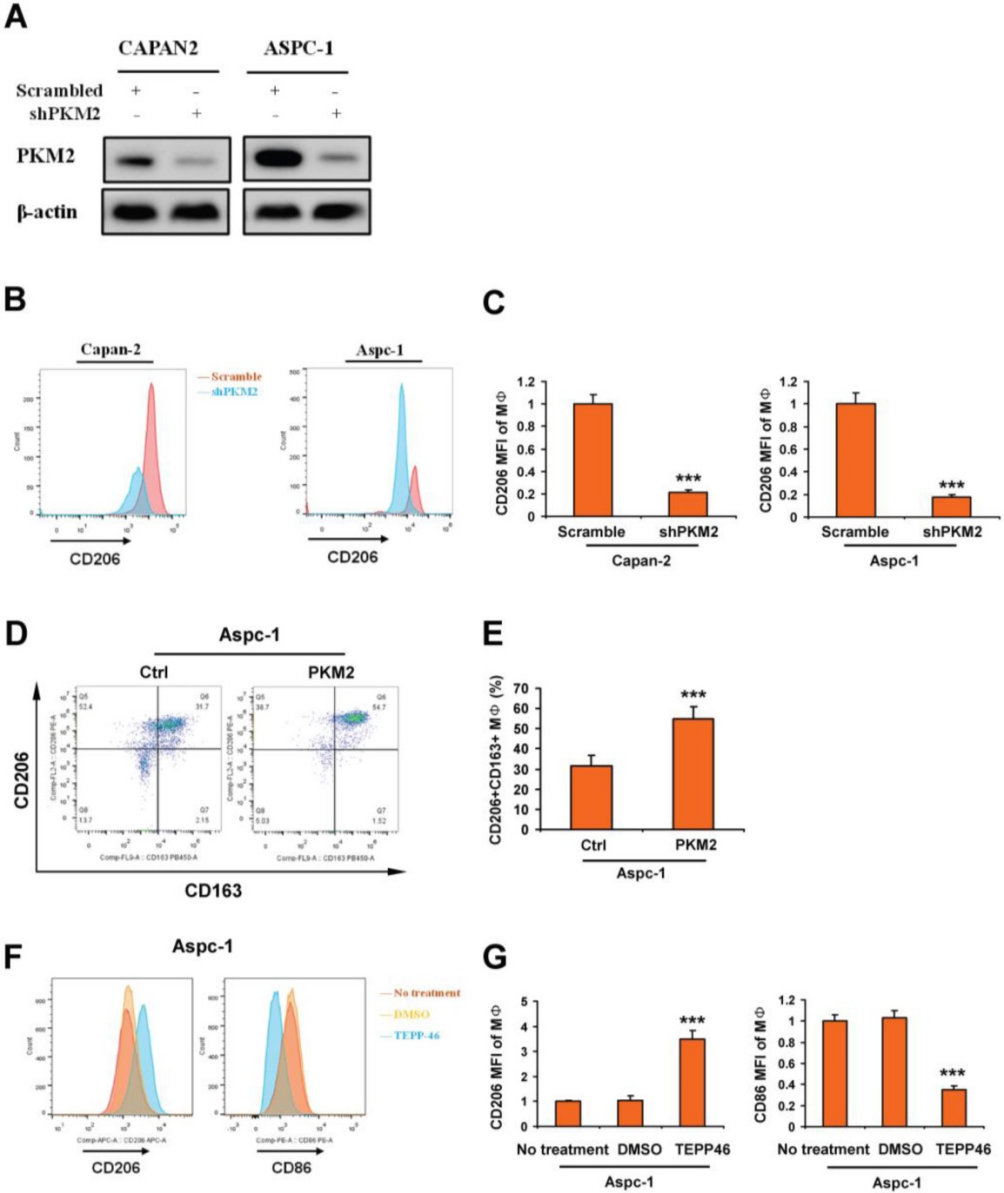
Overexpression of PKM2 in PDAC promotes macrophage toward M2 polarization. A. Images indicated the effects of PKM2 knockdown in CAPAN2 and ASPC-1 cells. B and C. PBMC-derived macrophages (MΦ) were co-cultured with PDAC cells with PKM2 kncokdown. And then the expression of CD206 in macrophages was evaluated by detecting the mean fluorescence intensity (MFI) using FACS. D-G. MΦ was co-cultured with PDAC cells with PKM2 agonist treatment. And then the expression of CD206, CD163 and CD86 in macrophages was evaluated by detecting the mean fluorescence intensity (MFI) using FACS. Data, mean±SEM; *** p < 0.001.

To investigate whether PKM2 expression in PDAC was associated with macrophage polarization, knockdown of PKM2 was performed in CAPAN2 and ASPC-1. Results of Western blot showed that the expression of PKM2 was obviously inhibited (Figure 4A). Macrophages were derived from human monocytes isolated from fresh blood, and then were co-cultured with CAPAN2 and ASPC-1 cells with PKM2 knockdown. We detected the expression of CD206 in macrophages by FACS. By analyzing the mean fluorescence intensity (MFI), we found that knockdown of PKM2 in PDAC cells significantly inhibited the expression of CD206 (Figure 4B-C). Next, PBMC-derived macrophages (MΦ) were co-cultured with APSC-1 with overexpression of PKM2. The expression of CD163 and CD206 on MΦ was detected by FACS. Our results showed that the number of CD206+CD163+ MΦ was significantly increased (Figure 4D-E). In addition, MΦ was co-cultured with APSC-1 cells treated with PKM2 agonist TEPP-46. Subsequently, the expression of CD206 and CD86 on macrophages was evaluated. We showed that CD206 levels were significantly enhanced by PDAC cells treated by TEPP-46, whereas CD86 levels were significantly decreased (Figure 4F-G). The results from TEPP-46 treatment were consistent with the experimental results from overexpression of PKM2. These results revealed that increased expression of PKM2 in PDAC promoted macrophage toward M2polarization.

## Discussion

**Figure 5:**
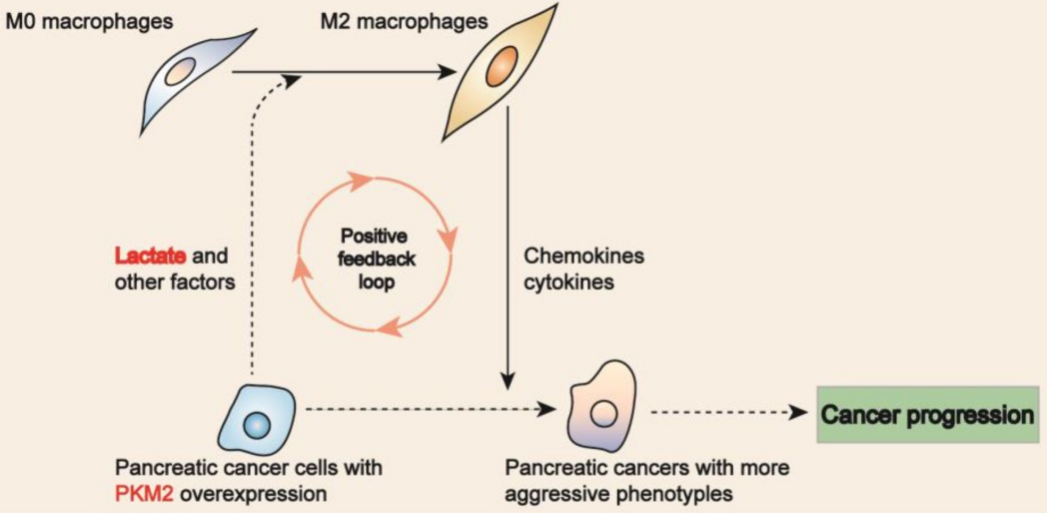
Proposed model links M2 macrophages to PKM2 in PDAC. PDAC cells with PKM2 overexpression resulted in lactate secretion, which then favors M2 macrophages in phenotype. Inversely, M2 macrophages 're-educate' the neoplastic cells toward a more aggressive phenotype by secreting bioactive factors.

In this study, we evaluated the biological significance of M2 macrophages in PDAC. Our data showed that M2 macrophages and PKM2 overexpression were independent prognostic factors for PDAC. Moreover, our data also indicated that PKM2 and CD206 expression had a synergic effect on facilitating PDAC progression. In co-culture system of macrophages and PDAC cells, we showed PDAC cells with PKM2 overexpression promoted macrophages toward M2 type. Since lactate, the product of PKM2 in glucose metabolism, could facilitate macrophages polarization toward M2 macrophage, which could inversely affect the neoplastic cells via the secretion of bioactive factors, leading to a more aggressive phenotype^14^, ^15^, we postulated that PKM2-resultant lactate favors M2 macrophages, which then inversely affect the cancers cells so as to promote the disease progression (Figure 5).

As the most abundant inflammatory cells in pancreatic microenvironment, TAMs were gaining more and more interests in the recent years. Zhang et al. showed that elevated M2 macrophages in lung cancer were associated with poor prognosis 16. Several studies had indicated that M2 macrophages promoted tumor progression by enhancing proliferation, metastasis, chemoresistance as well as angiogenesis of human solid tumors 17-18. Mechanistically, M2 macrophages favors chemoresistence via upregulating epithelial-mesenchymal transition (EMT) through the activation of transforming growth factor-β (TGF-β) signaling in various human solid malignancies^19^. In PDAC, some previous studies found that M2 macrophages could enhance EMT through toll-like receptor 4 (TLR4)/interleukin- 10 (IL-10) signaling, resulting in enhanced cell proliferation and migration 8, 20. In this study, we also showed that M2 macrophages within the pancreatic microenvironment were pro-tumoral and responsible for a poor prognosis. Despite of these advancements, little was known about how M2 macrophages were transformed in the lethal disease. In the present study, we found that PKM2 expression was positively correlated with M2 macrophages infiltration, suggesting that PKM2 overexpression in PDAC cells might facilitate M2 macrophages polarization.

Warburg effect was proposed by Otto Warburg in 1924, which put that cancer cells convert glucose to lactate even in the presence of oxygen so as to obtain essentialsubstrate and energy 21. Pyruvate kinase (PK) is arate-limiting enzyme in the glycolytic process, which functions to catalyze the production of pyruvate and adenosine triphosphate (ATP) from phosphoenolpyruvate (PEP) and adenosine diphosphate (ADP) 22. Pyruvate kinase M2 (PKM2), an indispensable member of PK family, was elevated in various human malignancies associating with proliferation, EMT, angiogenesis as well as chemoresistance in various human cancers 23-29. Recently, O'Neill, L.A and colleagues found that lactate facilitate M2 macrophages via the activation of HIF-1^9^, suggesting that PKM2 might involve in M2 macrophage polarization by promoting lactate production. Since our data revealed a positive correlation between PKM2 expression and M2 macrophages infiltration in PDAC, the singling axis “PKM2 -lactate in cancer cells to HIF-1-M2 macrophages” might help to explain the interactions between PKM2 and M2 macrophages in PDAC patients.

In fact, there were also limitations of our study. First, we concluded the positive correlations between PKM2 and M2 macrophages in PDAC merely based on the statistical analysis without further cellular and molecular function studies *in vitro* and *in vivo*. In addition, we set no further evidence to reveal the detailed crosstalk between PKM2 expression and M2 macrophage infiltration in PDAC. Finally, we simply indicated that the combination of PKM2 and M2 macrophage could facilitate PDAC progression with unknown mechanism. Obviously, the comprehensive mechanisms studies might help to reveal novel therapeutic targets for the disease.

## Conclusion

The study illustrated the biological significance of macrophage in PDAC, which showed that M2 macrophage highly infiltrated in PDAC and responsible for a poor survival. The data also showed that PKM2 was overexpression in PDAC cancer cells and functioned as independent prognostic factor, and positively correlated with M2 macrophage infiltration in PDAC. Moreover, our data showed that they were internal correlated and had a synergic effect on PDAC progression. We believed that a more comprehensive understanding toward the crosstalk between the two factors might reveal novel therapeutic targets for the lethal disease.

## Figures and Tables

**Table 1:** Baseline features of the patients

Factor		Number
**Gender**	Male	51
	Female	26
**Age**	≥60	51
	<60	26
**T stage**	T3	57
	≤T2	20
**N stage**	N0	35
	N1	42
**M stage**	M0	73
	M1	4
**TMN stage**	≥II	63
	I	14
**Tumor sites**	Head/Neck	40
	Body/Tail	37
**Nerve invasion**	Yes	49
	No	28
**Vascular invasion**	Yes	10
	No	67
**Nuclear grade**	III	26
	≤II	51

**Table 2:** PKM2 and CD206 expression in the cancerous tissues and the adjacent tissues

	Number	PKM2		P value	CD206		P value
		Negative	Positive		Negative	Positive	
**Cancerous tissues**	77	37	40	p<0.05	35	42	p<0.05
**Adjacent tissues**	77	57	20		54	23	

**Table 3:** Correlation between PKM2, CD206 and clinicopathologic features of PDAC

Factor	PKM2		P value	CD206		P value
	Negative	Positive		Negative	Positive	
**Gender**						
Male	24	27	0.807	23	28	0.93
Female	13	13		12	14	
**Age**						
≥60	27	24	0.2291	25	26	0.379
<60	10	16		10	16	
**T stage**						
T3	29	28	0.402	25	32	0.635
≤T2	8	12		10	10	
**N stage**						
N0	18	17	0.298	10	25	0.007
N1	19	23		25	17	
**M stage**						
M0	36	37	0.664	33	40	1
M1	1	3		2	2	
**TMN stage**						
≥II	24	39	0.0002	27	36	0.331
I	13	1		8	6	
**Tumor sites**						
Head/Neck	17	22	0.137	18	22	0.934
Body/Tail	20	18		17	20	
**Nerve invasion**						
Yes	22	20	0.698	25	24	0.194
No	15	20		10	18	
**Vascular invasion**						
Yes	9	1	0.012	2	8	0.164
No	28	39		33	34	
**Nuclear grade**						
III	12	24	0.056	13	13	0.567
≤II	35	16		22	29	

**Table 4:** the correlation between PKM2 and CD206 in PDAC

Tumor tissues	PKM2 expression				Correlation coefficient	P value
	0	1	2	3		
**CD206(0)**	9	1	2	0	rs=0.175	0.03
**CD206(1)**	2	12	7	2		
**CD206(2)**	3	9	22	0		
**CD206(3)**	0	1	1	6		

**Table 5:** Univariate and multivariate survival analysis of PDAC patients

Factor	OS median (range)	Univariate analysis				Multivariate analysis		
		HR	95%CI	P value		HR	95%CI	P value
**Gender**								
Male	19.0(1.0-47.0)	1.054	0.555-2.004	0.871				
Female	20.0(3.0-46.0)	1						
**Age**								
≥60	22.0(1.0-47.0)	0.677	0.365-1.254	0.215				
<60	15.5(2.0-46.0)	1						
**T stage**								
T3	17.8(1.7-47.6)	1.778	0.822-3.845	0.144				
≤T2	23.0(3.0-46.0)	1						
**N stage**								
N0	22.3(2.9-47.6)	1.279	0.694-2.357	0.431				
N1	17.(1.7-45.8)	1						
**M stage**								
M0	18.4(1.7-47.6)	0.658	0.175-3.005	0.658				
M1	23.2(12.4-40.3)	1						
**TMN stage**								
≥II	15.6(1.7-47.6)	3.052	1.087-8.567	0.034		2.899	1.731-4.855	<.0001
I	31.1(12.6-46.5)	1				1		
**Primary tumor location**								
Head and Neck	16.4(1.7-47.6)	1.204	0.657-2.209	0.548				
Body and Tail	23.1(2.9-46.4)	1.000						
**Nerve invasion**								
Yes	13.6(1.7-46.5)	2.171	1.089-4.328	0.028		1.592	0.989-2.560	0.055
No	23.7(3.1-47.6)	1.000				1.000		
**Vascular invasion**								
Yes	7.9(3.1-46.5)	1.926	1.198-3.095	0.007		1.583	0.984-2.546	0.058
No	22.3(1.7-47.6)	1.000				1.000		
**Nuclear grade**								
III	12.1(1.7-44.6)	2.374	1.290-4.368	0.005		2.519	1.534-4.137	0.000
<II	23.2(4.0-47.6)	1.000				1.000		
**PKM2**								
Positive	12.5(1.7-46.5)	2.326	1.428-3.788	0.001		2.117	1.309-3.426	0.002
Negative	23.7(2.9-47.6)	1.000				1.000		
**CD206**								
Positive	9.4(1.7-46.5)	1.861	1.159-2.988	0.010		1.595	0.992-2.566	0.054
Negative	23.2(2.9-47.6)	1.000				1.000		
**PKM/CD206**								
PKM+/CD206+	7.3(1.7-46.5)	2.558	1.556-4.205	0.000		3.349	1.956-5.735	0.001
All others	23.1(2.9-47.6)	1.000				1.000		
